# Noninvasive cardiac radioablation for ventricular tachycardia: dosimetric comparison between linear accelerator- and robotic CyberKnife-based radiosurgery systems

**DOI:** 10.1186/s13014-023-02370-w

**Published:** 2023-11-10

**Authors:** Ching-Yu Wang, Li-Ting Ho, Lian-Yu Lin, Hsing-Min Chan, Hung-Yi Chen, Tung-Lin Yu, Yu-Sen Huang, Sung-Hsin Kuo, Wen-Jeng Lee, Jenny Ling-Yu Chen

**Affiliations:** 1 Division of Radiation Oncology, Department of Oncology, National Taiwan University Hospital, Taipei, Taiwan; 2 Division of Cardiology, Department of Internal Medicine, National Taiwan University College of Medicine and Hospital, Taipei, Taiwan; 3 Department of Radiation Oncology, Fu-Jen Catholic University Hospital, Taipei, Taiwan; 4 Department of Radiation Oncology, National Taiwan University Cancer Center, No. 57, Ln. 155, Sec. 3, Keelung Rd., Taipei, 106 Taiwan; 5 Department of Medical Imaging, National Taiwan University Hospital, No. 7, Chung-Shan S. Rd., Taipei, 100 Taiwan; 6 Department of Radiology, National Taiwan University College of Medicine, Taipei, Taiwan

**Keywords:** CyberKnife, Linear accelerator, Cardiac radioablation, Ventricular tachycardia

## Abstract

**Background:**

Few dosimetric comparisons have been published between linear accelerator (LA)-based systems and CyberKnife (CK)-based robotic radiosurgery systems for cardiac radio-ablation in ventricular tachycardia. This study aimed to compare the dosimetry of noninvasive cardiac radio-ablation deliverable on LA with that on CK.

**Methods:**

Thirteen patients who underwent noninvasive cardiac radio-ablation by LA were included. The prescribed dose was 25 Gy in 1 fraction, and the average planning target volume was 49.8 ± 31.0 cm^3^ (range, 14.4–93.7 cm^3^). CK plans were generated for comparison.

**Results:**

Both the CK and LA plans accomplished appropriate dose coverage and normal tissue sparing. Compared with the LA plans, the CK plans achieved significantly lower gradient indices (3.12 ± 0.71 vs. 3.48 ± 0.55, *p* = 0.031) and gradient measures (1.00 ± 0.29 cm vs. 1.17 ± 0.29 cm, *p* < 0.001). They had similar equivalent conformity indices (CK vs. LA: 0.84 ± 0.08 vs. 0.87 ± 0.07, *p* = 0.093) and maximum doses 2 cm from the planning target volume (PTV) in any direction (CK vs. LA: 50.8 ± 9.9% vs. 53.1 ± 5.3%, *p* = 0.423). The dosimetric advantages of CK were more prominent in patients with a PTV of ≤ 50 cm^3^ or a spherical PTV. In patients with a PTV of > 50 cm^3^ or a non-spherical PTV, the LA and CK plans were similar regarding dosimetric parameters. CK plans involved more beams (232.2 ± 110.8 beams vs. 10.0 ± 1.7 arcs) and longer treatment times (119.2 ± 43.3 min vs. 22.4 ± 1.6 min, *p* = 0.007).

**Conclusions:**

Both CK and LA are ideal modalities for noninvasive cardiac radio-ablation. Upfront treatment should be considered based on clinical intent.

## Background

Noninvasive cardiac radio-ablation by stereotactic ablative radiotherapy represents an alternative treatment modality for ventricular tachycardia (VT). It effectively reduced the VT burden and improved the quality of life for patients who are refractory to medical therapy or catheter ablation or who cannot tolerate it [1, 2]. Currently, the linear accelerator (LA)-based system and the CyberKnife (CK) robotic radiosurgery system are used for noninvasive cardiac radio-ablation. Notably, several studies on the LA-based system from the USA, Asia, and Europe applied coplanar and non-coplanar volumetric-modulated arc radiotherapy (VMAT) to generate a highly conformal ablative radiation dose to the VT substrate [3–9]. Meanwhile, studies on the CK robotic radiosurgery system from the USA and Europe used a six-dimensional robotic arm, multiple non-coplanar small beams, and a continuous image-tracking system to deliver ablative radiation doses accurately [10–13].

Noninvasive cardiac radio-ablation for VT is a new and unique challenge for clinicians and radiation physicists. One case report presented the dosimetric difference between the VT substrate’s LA- and CK-based systems at the anterior basal heart [14]. The report concluded that CK was superior to the LA-based planning system for a steeper dose gradient and better real-time target tracking. The RAVENTA benchmark study included three patients’ plans using LA- and CK-based systems from five academic centers [15]. They demonstrated that VMAT plans had steeper dose gradients in the high-dose region, while the CK plans had smaller low-dose regions. They concluded that plans from both systems were considered deliverable based on the internal guidelines and protocols for SBRT and noninvasive cardiac radio-ablation [16].

Few dosimetric comparisons have been published between the LA-based and CK-based robotic radiosurgery systems for cardiac radio-ablation. Therefore, this study aimed to compare the dosimetry of noninvasive cardiac radio-ablation deliverables on CK with LA systems and discuss related clinical issues.

## Methods

### Study design and patients

This study was approved by the National Taiwan University Hospital Research Ethics Committee (approval number: 201804026RINC). All procedures in this study followed the ethical standards of the institutional and/or national research committee and the 1964 Declaration of Helsinki and its later amendments or comparable ethical standards. Written informed consent was obtained from all patients. Thirteen patients who underwent noninvasive cardiac radio-ablation for VT by an LA-based system using approved VMAT plans at our institution were included between March 2018 and March 2022.

### Treatment protocol

**Fig. 1:**
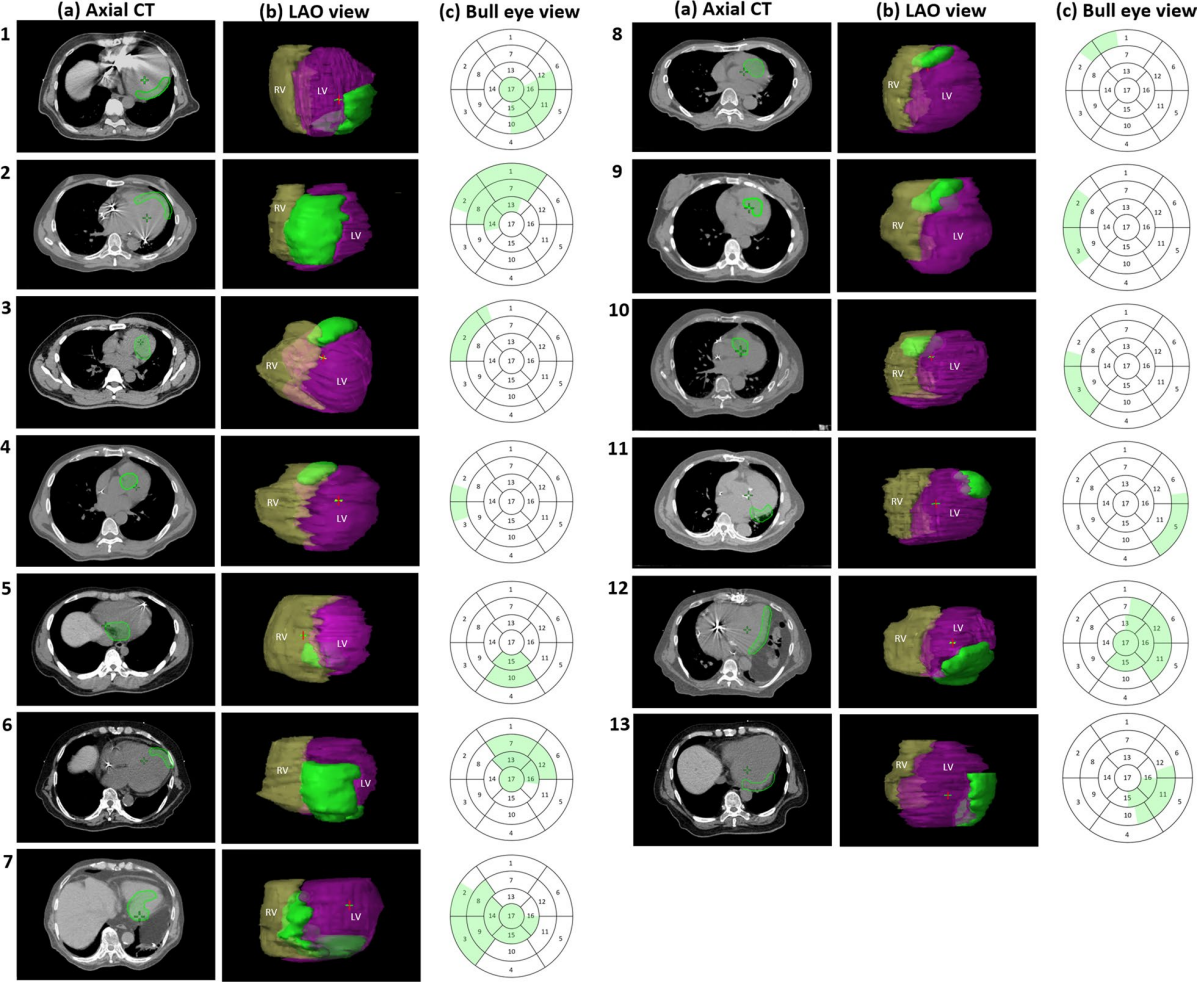
Demonstrations of the 13 target volumes. Complex-shaped target volumes are shown at the left ventricle (LV)’s basal, middle, and apical areas. **a** Axial view of the planning target volume (PTV) as a green-colored wash area. **b** Three-dimensional representation of the PTV (green), LV (pink), and right ventricle (RV, yellow) on left anterior oblique (LAO) views. **c** Bull’s eye view displaying the green target area in the 17-segment American Heart Association model

Stereotactic body radiation therapy (SBRT) simulations, substrate identification, and contour delineation have been previously described [5, 17]. All patients in this study underwent standard SBRT simulation while immobilized in an individualized vacuum bag (BodyFIX; Elekta, Stockholm, Sweden), which limited diaphragmatic motion through external abdominal compression. Axial images (1-mm slices) were obtained. Cardiac magnetic resonance imaging, diagnostic dual-energy computed tomography (CT), and three-dimensional electroanatomic maps defined the treatment target volume. The average planning target volume (PTV) was 49.8 ± 31.0 cm^3^ (range, 14.4–93.7 cm^3^), and the prescribed dose was 25 Gy in 1 fraction. The targets were heterogeneous concerning shape and location; involved complex-shaped target volumes at the left ventricular (LV) basal, middle, or apical areas; and were surrounded by non-identical critical structures, as shown in Fig. 1. The bull’s eye view displayed the target area in the 17-segment mode proposed by the American Heart Association [18, 19].

### Treatment planning

The clinical LA VMAT plans were generated initially using the treatment planning system (TPS) (Eclipse, version 15.5; Varian Medical Systems, Palo Alto, CA, USA), with 6- or 10-MV flattening filter-free photon beams, multiple coplanar and non-coplanar partial arcs, and the VMAT technique. The dose calculation algorithm was anisotropic analytical algorithm (AAA). The prescribed isodose was adequate to cover at least 95% of the target. The dose constraints for single-fraction SBRT for organs at risk (OARs) were adopted from the American Association of Physicists in Medicine Task Group 101 (TG101) as follows: skin, 26 Gy; rib, 30 Gy; trachea/large bronchus, 20 Gy; spinal cord, 14 Gy; stomach, 12.4 Gy; the esophagus, 15.4 Gy; lungs (volume of at least 1500 cm^3^), < 7 Gy; liver (volume of at least 700 cm^3^), < 9.1 Gy [20, 21].

**Fig. 2:**
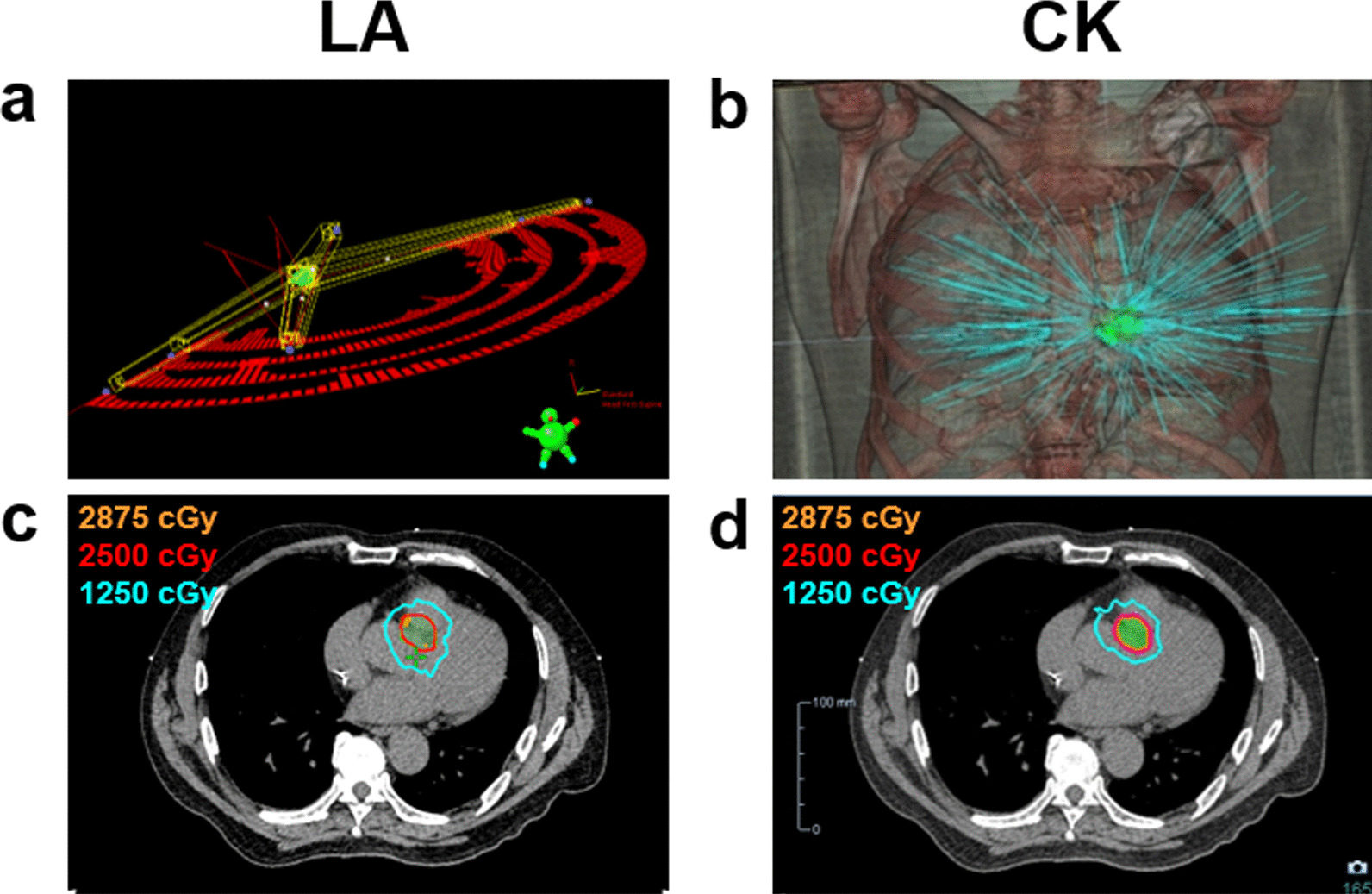
Example of noninvasive cardiac radio-ablation using the linear accelerator (LA) and CyberKnife (CK)-based radiosurgery systems. Patient No. 10 was a 61-year-old man with recurrent sustained ventricular tachycardia (VT) related to left apical ventricular hypertrophy refractory to antiarrhythmic medication and an implantable cardiac defibrillator. Electro-anatomical mapping revealed that the substrate originated from the apical junction of the right coronary commissure (RCC) and left coronary commissure (LCC). He underwent multiple sessions of CARTO-guided cardiac ablation of the apical junction of the LCC-RCC and the right ventricular outflow tract posterior septal area. He developed recurrent VT, and noninvasive cardiac radio-ablation was performed using an LA-based 6-MV flattening filter-free photon beam and stereotactic volumetric modulated arc radiotherapy technique to the posterior-septal wall at 25 Gy in 1 fraction. The VT burden was reduced after noninvasive cardiac radio-ablation, and the patient was followed up at the clinic. The CK plan was generated for dosimetric comparison. The homogeneity indices, conformity indices, gradient indices, gradient measures, and maximum doses at 2 cm from the planning target volume (PTV) of the LA and CK plans are shown in Table 1. The beam arrangement is shown in the upper panels of the figure. **a** Four coplanar arcs and three non-coplanar arcs are presented in the LA plan. **b** A total of 253 non-coplanar beams are presented in the CK plan. The isodose curves are shown in the lower panel for the LA plan (**c**) and the CK plan (**d**). Green areas indicate the PTV. The orange, red, and blue lines represent the isodose curves at 2875, 2500, and 1250 cGy, respectively

The planning CT dataset and pre-drawn structures in the Eclipse TPS were transferred to the CyberKnife M6 TPS (Precision version 3.3.1.2; Accuray, Sunnyvale, CA, USA) to generate CK plans for comparison. Experienced medical physicists performed planning in the CK TPS based on the planning directive without prior knowledge of the quality of the LA clinical plan. Specifically, these research plans were generated as if they were for clinical use rather than demonstrating superiority over the LA plan. For the IRIS mode, the treatments were planned with VOLO optimizer utilizing variable aperture IRIS which uses different sizes of the collimator to generate hundreds of non-isocentric beams in one path to spare all the critical organs near the target and fulfill the criteria as above, and the dose calculation algorithm was Ray-tracing with contour correction; for the InCise MLC mode frequently used to treat large and irregularly shaped targets with the advantage of shorter delivery time, the dose calculation algorithm was finite size pencil beam (FSPB). The 6-MV flattening filter-free beams were delivered from non-coplanar angles to improve the conformity of the radiation dose and reduce radiation damage to healthy tissues. An example of noninvasive cardiac radio-ablation using the CK- or LA-based systems with associated isodose curves is shown in Fig. 2.

### Plan comparison

All plans were planned for at least 95% coverage of the target volume with the prescribed dose. As previously described, the plans had to fulfill the predefined dose constraints for OARs. When there was a trade-off between OAR dosimetry and target coverage, dose constraints to the esophagus and stomach were prioritized over the PTV coverage. For a fair comparison between LA and CK, the CK plan’s coverage was normalized to that of the LA plan. For example, if the CK and LA plans of the same patient had coverage of 95.0% and 95.5%, the CK plan was normalized so that 95.5% of the PTV received the prescription dose, the same as in the counterpart LA plan.

Since DVH calculation on different TPS possessed potential uncertainties, dose distributions of CK plans were imported into Eclipse TPS for plan comparison [15, 22]. The quality indices used for plan comparison were the homogeneity index (HI), Paddick conformity index (CI), gradient index (GI), gradient measure (GM), and maximum dose 2 cm from the PTV in any direction (D_2cm_), regarding the standardization of terminology in stereotactic radiosurgery [23]. The HI was calculated as the maximum target dose divided by the prescription dose. The CI was calculated as TV_PIV_^2^/(TV × PIV), where TV represented the target volume; prescription isodose volume (PIV) represented the volume that received the prescription dose, whether within or outside the targets; and TV _PIV_ represented TV covered by the PIV. A perfect plan was defined to have a CI score of 1, and a plan CI of > 0.85 was considered ideal. GI was calculated as PV_50%_/PIV, where PV_50%_ represented the volume covered by 50% of the prescription dose. The GM defined the average distance between the 12.5-Gy equivalent spherical volume and the 25-Gy equivalent spherical volume. A smaller GI or GM value indicated a steeper dose gradient. D_2cm_ was used to assess the intermediate-to-low dose spillage outside the PTV.

The maximal doses to the spinal cord, stomach, esophagus, trachea/large bronchus, and coronary arteries (including the left anterior descending artery, right coronary artery, and left circumflex artery) were recorded for OAR dosimetry. The TG101 study also suggested that the heart volume receiving at least 16 Gy should be maintained at < 15 cm^3^ [19]. However, this was not possible during cardiac radio-ablation owing to the nature of the prescription dose and the target locations. In addition to the abovementioned parameters, the total volume of the heart receiving > 16 Gy, excluding the PTV (heart minus PTV), was also compared.

The number of beams and monitor units required to deliver the prescribed dose in the LA or CK plans was evaluated. The estimated treatment time for LA included both the treatment delivery time and a 15-min length for the cone beam CT (CBCT) imaging registration; according to the institutional SBRT protocol, at least three sets of CBCT scans (the first CBCT was for bony and soft tissue registration, the second was to confirm the correction before coplanar arcs irradiation, and the third was before the start of non-coplanar beams) were scheduled for each patient. The CK TPS reported the beam delivery time, which included both the beam-on time and time for location tracking.

For subgroup analysis, the targets were classified into large or small volumes, spherical or non-spherical, according to their volumes and shapes in three dimensions. Large targets were as PTV > 50 cm^3^ [24]. Spherical targets were shapes in three dimensions, being more or less round, with smooth borders, and recognizable decreasing areas in three dimensions from the central plane to the periphery [25].

### Statistical analyses

Two-tailed paired-sample t-tests were conducted to evaluate the statistical significance of the differences between the two modalities for the plan quality indices. All statistical analyses were performed using Statistical Package for Social Sciences for Windows (SPSS version 22.0; IBM, Armonk, NY, USA). Statistical significance was set at *p* ≤ 0.05*.*

## Results

**Fig. 3:**
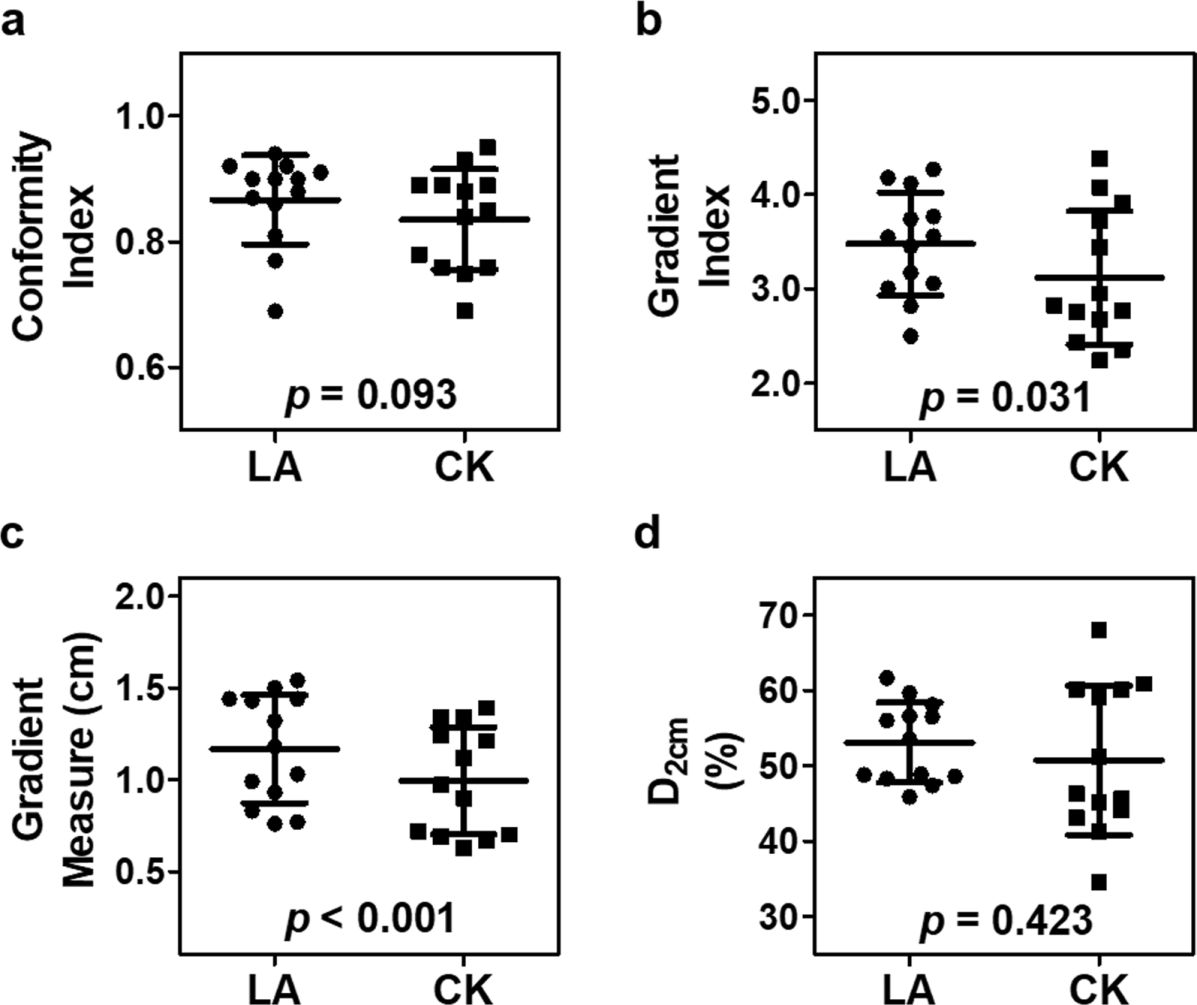
Dosimetric comparison between linear accelerator (LA) and CyberKnife (CK) plans. The conformity indices (**a**), gradient indices (**b**), gradient measures (**c**), and maximum doses 2 cm from the planning target volume in any direction (**d**) of the LA and CK plans are demonstrated. *p*-values for statistical comparisons are obtained using a paired Student’s t-test

The clinical goal of 95% PTV coverage was optimally achieved, except in patient No. 12, who had a large PTV of 93.7 cm^3^ located at the apical lateral wall of the LV adjacent to the stomach. The PTV coverage was traded off in both the LA and CK systems to reach the dose constraint of the stomach. The plan quality parameters are listed in Table 1 and Fig. 3. The HI ratio was significantly higher in the CK plans than in the LA plans (1.34 ± 0.06 vs. 1.24 ± 0.03, *p* < 0.001), reflecting that a higher maximal dose within target volumes was usually tolerable for CK-based radiosurgery plans. The CK and LA plans achieved acceptable CI ratios, with no significant difference (CK vs. LA: 0.84 ± 0.08 vs. 0.87 ± 0.07, respectively, *p* = 0.093).

**Table 1:** Comparison of plan quality parameters between the LA- and CK-based systems for 13 clinical cases

No	PTV (cm^3^)	Coverage (%)	HI (Ratio)		CI (Ratio)		GI (Ratio)		GM (cm)		D_2cm_ (%)	
			LA	CK	LA	CK	LA	CK	LA	CK	LA	CK
1	52.1	95.6	1.19	1.30	0.81	0.69	4.27	3.72	1.44	1.24	59.7	51.2
2	80.5	97.2	1.24	1.32	0.77	0.76	4.12	4.38	1.54	1.39	58.1	60.9
3	23.9	95.0	1.25	1.28	0.90	0.89	3.56	2.43	1.03	0.69	61.7	41.3
4	14.4	95.4	1.21	1.28	0.90	0.95	4.18	2.67	0.99	0.70	48.8	34.5
5	34.4	95.0	1.21	1.23	0.91	0.89	3.45	2.77	1.18	0.97	56.6	45.2
6	83.4	95.5	1.26	1.30	0.86	0.78	3.74	3.91	1.50	1.34	56.5	59.1
7	92.6	95.0	1.29	1.33	0.90	0.84	3.17	2.76	1.44	1.21	56.0	60.1
8	19.6	95.0	1.22	1.43	0.92	0.85	3.06	2.95	0.77	0.67	47.4	44.1
9	26.1	95.0	1.26	1.40	0.92	0.89	3.01	2.83	0.83	0.72	48.6	45.6
10	24.2	95.0	1.28	1.37	0.94	0.93	2.82	2.36	0.76	0.63	45.9	46.3
11	24.6	95.1	1.24	1.39	0.87	0.88	3.55	3.44	0.93	0.90	48.3	43.1
12	93.7	69.5	1.23	1.43	0.69	0.75	2.50	2.24	1.32	1.12	48.9	68.0
13	77.6	95.3	1.21	1.37	0.88	0.76	3.77	4.08	1.43	1.34	53.5	60.2
Mean ± SD	49.8 ± 31.0	93.0 ± 7.2	1.24 ± 0.03	1.34 ± 0.06	0.87 ± 0.07	0.84 ± 0.08	3.48 ± 0.55	3.12 ± 0.71	1.17 ± 0.29	1.00 ± 0.29	53.1 ± 5.3	50.8 ± 9.9
*p*-value*			< 0.001		0.093		0.031		< 0.001		0.423	

*CI* conformity index, *CK* CyberKnife, *D*_2cm_ maximum dose 2 cm from the PTV in any direction, *GI* gradient index, *GM* gradient measure, *HI* homogeneity index, *LA* linear accelerator, *PTV* planning target volume

*Significance is tested using the paired-sample t-test, with *p* < 0.05 indicating statistical significance

The CK plans achieved significantly lower GI ratios (3.12 ± 0.71 vs. 3.48 ± 0.55, *p* = 0.031) and GM values (1.00 ± 0.29 cm vs. 1.17 ± 0.29 cm, *p* < 0.001) than the LA plans, indicating that the CK plans provided a steeper dose gradient benefit. The D_2cm_ was comparable between the LA and CK plans (CK vs. LA: 50.8 ± 9.9% vs. 53.1 ± 5.3%, respectively, *p* = 0.423), indicating that in both systems, the radiation dose could be decreased to approximately half values at 2 cm from the PTV.

The dosimetric parameters of the OARs are listed in Table 2. The maximal doses to the esophagus, stomach, spinal cord, trachea, large bronchus, and coronary artery were similar between the CK and LA plans. The volumes of the normal heart receiving ≥ 16 Gy, the normal heart’s mean dose, and the normal lung receiving ≥ 7 Gy were similar between the CK and LA plans. These results suggested that the OARs were well protected with the two systems.

**Table 2:** Comparison of dosimetric parameters between the LA- and CK-based systems for 13 clinical cases

	LA-based system		CK-based system		*p*-value*
	Mean ± SD	Range	Mean ± SD	Range	
Esophagus max, Gy	7.3 ± 4.5	2.7–14.9	6.8 ± 4.6	1.9–15.1	0.193
Stomach max, Gy	6.6 ± 5.2	0.5–12.4	7.3 ± 5.1	0.2–12.2	0.120
Spinal cord max, Gy	2.7 ± 1.2	1.3–4.7	2.5 ± 0.8	1.2–4.0	0.346
Trachea/large bronchus, Gy	2.5 ± 3.8	0.3–12.2	2.4 ± 3.0	0.2–9.4	0.927
Lung V7, cm^3^	51.2 ± 51.3	5.2–185.1	47.2 ± 56.5	2.3–193.2	0.263
(Heart–PTV) V16, cm^3^	13.6 ± 3.5	7.7–20.5	13.9 ± 6.0	4.7–25.4	0.765
(Heart–PTV) mean dose, Gy	3.3 ± 1.2	1.8–6.1	2.6 ± 0.8	1.7–4.9	0.282
Coronary artery max, Gy	20.0 ± 5.2	8.4–26.3	19.6 ± 7.0	7.5–28.5	0.633

*CK* CyberKnife, *LA* linear accelerator; max, maximal dose, *PTV* planning target volume, *SD* standard deviation, *V7* volume (cm^3^) receiving ≥ 7 Gy, *V16* volume (cm^3^) receiving ≥ 16 Gy

*Significance is tested using the paired-sample t-test, with *p* < 0.05 indicating statistical significance

The treatment delivery parameters are listed in Table 3. An average of 232.2 non-coplanar beams in the CK system and a median of 10 arcs (six coplanar arcs and four non-coplanar arcs) in the LA system were designed for one fully qualified plan. Compared with the LA plans, the CK plans had significantly longer treatment times (22.4 ± 1.6 min vs. 119.2 ± 43.3 min, *p* = 0.007).

**Table 3:** Treatment delivery parameters of the LA- and CK-based systems for 13 clinical cases

	LA-based system		CK-based system	
	Median ± SD	Range	Mean ± SD	Range
Number of partial arcs for LA or beams for CK*	Coplanar: 6.0 ± 1.3 Non-coplanar: 4.0 ± 0.8 Total: 10.0 ± 1.7	Coplanar: 4.0–9.0 Non-coplanar: 2.0–4.0 Total: 7.0–13.0	232.2 ± 110.8	38.0–308.0
Monitor units	9739.4 ± 1903.3	6431.0–12448.0	39,993.0 ± 11,786.0	12,433.0–54574.0
Treatment time estimates (min)	22.4 ± 1.6	20.2–24.1	119.2 ± 43.3	46.0–159.0

*CK* CyberKnife, *LA* linear accelerator, *SD* standard deviation

*Both coplanar and non-coplanar arcs are used in the LA system, while only non-coplanar beams are used in the CK system

The dosimetric comparison between large (PTV > 50 cm^3^, n = 6) and small (PTV ≤ 50 cm^3^, n = 7) target volumes for subgroup analyses is shown in Table 4. The dosimetric advantages of CK providing steeper dose gradients were prominent in patients with small target volumes, as evidenced by the lower GI ratios (2.78 ± 0.36 vs. 3.38 ± 0.46, *p* = 0.028), GM values (0.76 ± 0.13 cm vs. 0.93 ± 0.15 cm, *p* = 0.007), and D_2cm_ values (42.9 ± 4.1% vs. 51.0 ± 5.8%, *p* = 0.027). Meanwhile, the dosimetric advantages of CK were less evident in patients with large target volumes, as evidenced by the lower GM values (1.28 ± 0.10 cm vs. 1.45 ± 0.07 cm, *p* < 0.001) but similar GI ratios (CK vs. LA: 3.52 ± 0.83 vs. 3.60 ± 0.66, respectively, *p* = 0.619) and D_2cm_ values (CK vs. LA: 59.9 ± 5.4% vs. 55.5 ± 3.8%, respectively, *p* = 0.273). There was no difference in the estimated treatment time between large and small target volumes in both the LA system (small vs. large: 21.7 ± 1.8 vs. 23.2 ± 0.8 min, respectively, *p* = 0.072) and the CK system (small vs. large: 120.2 ± 18.1 vs. 118.2 ± 37.4 min, *p* = 0.907).

**Table 4:** Subgroup analysis between the linear accelerator (LA)- and CyberKnife (CK)-based systems

	PTV ≤ 50 cm^3^ (n = 7)			PTV > 50 cm^3^ (n = 6)		
	LA	CK	*p*-value^†^	LA	CK	*p*-value^†^
CI (ratio)	0.91 ± 0.02	0.90 ± 0.03	0.551	0.82 ± 0.08	0.77 ± 0.05	0.129
GI (ratio)	3.38 ± 0.46	2.78 ± 0.36	0.028	3.60 ± 0.66	3.52 ± 0.83	0.619
GM (cm)	0.93 ± 0.15	0.76 ± 0.13	0.007	1.45 ± 0.07	1.28 ± 0.10	< 0.001
D_2cm_ (%)	51.0 ± 5.8	42.9 ± 4.1	0.027	55.5 ± 3.8	59.9 ± 5.4	0.273
Treatment time estimates (min)	21.7 ± 1.8	120.2 ± 18.1	< 0.001	23.2 ± 0.8	118.2 ± 37.4	0.002

	Spherical PTV (n = 6)			Non-spherical PTV (n = 7)		
	LA	CK	*p*-value^†^	LA	CK	*p*-value^†^
CI (ratio)	0.91 ± 0.01	0.90 ± 0.04	0.515	0.83 ± 0.07	0.78 ± 0.06	0.139
GI (ratio)	3.35 ± 0.50	2.63 ± 0.23	0.029	3.59 ± 0.60	3.51 ± 0.76	0.531
GM (cm)	0.93 ± 0.17	0.73 ± 0.12	0.006	1.37 ± 0.21	1.22 ± 0.17	0.001
D_2cm_ (%)	51.5 ± 6.2	42.9 ± 4.4	0.046	54.4 ± 4.4	57.5 ± 8.0	0.395

Values are presented as the mean ± standard deviation

*CI* conformity index, *D*_2cm_ maximum dose 2 cm from the planning target volume in any direction, *GI* gradient index, *GM* gradient measure, *PTV* planning target volume

^†^Significance is tested using the paired-sample t-test, with *p* < 0.05 indicating statistical significance

Additional analysis was conducted for dosimetric parameters by stratifying the PTV shape into spherical (n = 6) or non-spherical (n = 7). The benefits of CK plans for treating spherical targets were prominent, as manifested by lower GI ratios (2.63 ± 0.23 vs. 3.35 ± 0.50, p = 0.029), GM ratios (0.73 ± 0.12 vs. 0.93 ± 0.17, p = 0.006), and D_2cm_ values (42.9 ± 4.4% vs. 51.5 ± 6.2%, *p* = 0.046). When treating non-spherical PTVs, the LA plans achieved equivalent dosimetric advantages, as supported by similar GI ratios (CK vs. LA: 3.51 ± 0.76 vs. 3.59 ± 0.60, respectively, *p* = 0.531) and D_2cm_ values (CK vs. LA: 57.5 ± 8.0% vs. 54.4 ± 4.4%, respectively, *p* = 0.395).

## Discussion

There have been few dosimetric comparisons between the LA-based and CK-based robotic radiosurgery systems for cardiac radio-ablation. This study found that the LA and CK systems are suitable for noninvasive cardiac radio-ablation, with adequate dose coverage and appropriate normal tissue sparing. CK plans provided dosimetric advantages of steeper dose gradients in patients with small target volumes (PTV ≤ 50 cm^3^) or spherical PTVs. Meanwhile, CK plans required longer treatment times.

The present study demonstrated complex-shaped target volumes at the LV basal, middle, or apical areas surrounded by non-identical critical structures, indicating noninvasive cardiac radio-ablation heterogeneity and dosimetric challenges. The dose distribution of homogeneity and gradient measures were significantly more favorable in the CK plans than in the LA plans, especially in patients with small target volumes and spherical PTVs. This suggested a much sharper and rapid dose fall-off of the margin from target volumes and a more centralized uniform radiation dose within target volumes in CK plans. These results align with the published case report establishing the dosimetric benefit of using the CK system to treat non-ischemic basal VT with a small and spherical PTV (31.8 cm^3^) [14].

The RAVENTA trial demonstrated three benchmark cases with PTVs of 36.5 cc, 62.4 cc, and 52.8 cc [15]. The present study parallels their study regarding dose distribution and conformity values. The RAVENTA trial showed that the dose distributions were more axial in VMAT plans and more spherical in CK plans, and a trend toward higher CI ratios in the LA-based VMAT plans (LA 0.87 vs. CK 0.79), which following our distribution shown in Fig. 2 and the CI ratio results (LA 0.87 vs. CK 0.84). The GI advantage of CK plans in the present study was merely seen in the RAVENTA trial. In their series, the LA-based VMAT plans had steeper dose gradients in the high-dose region, while the CK plans had smaller low-dose regions. This may partly be related to their series’ target volumes and cardiac substructures constraint. The dosimetric advantages of CK were less evident in our series in patients with PTV > 50 cm^3^ showing similar GI ratios (CK 3.52 vs. LA 3.60). Moreover, the RAVENTA trial adopted dose constraints to cardiac substructures, including the aorta, the left coronary arteries, the superior vena cava, and the left atrium, which are not implemented in our study. The trade-offs between the gradient dose fall-off and OAR sparing varied from plan to plan, depending on individual planners, different institutions, and varied clinical situations [26, 27]. In our series, we observed that LA plans exhibited dosimetric superiority in thin and flat targets in the apical LV lateral wall, as shown in patients No. 6 and No. 13. This implies that treatment systems should be considered based on PTV volumes, locations, and shapes.

Literature has shown that for stereotactic radiotherapy, different dose calculation approaches like AAA, ray-tracing, or pencil beam algorithms may have differences regarding dose distributions, with the largest differences in the lung encompassing high tissue heterogeneities[28]. AAA algorithm accounts for changes in electron transport and volume scatter with improved calculation results, especially in regions of high heterogeneities [29], thus may be more reliable in circumstances where VT targets overlapping lung regions, while both the AAA algorithm and the pencil beam algorithm are accurate enough for cases where VT targets reside within the heart, where almost no heterogeneities are present. In the RAVENTA benchmark study, the dosimetric accuracy was tested during patient-specific quality assurance using an electronic portal imaging device (EPID) for VMAT plans and PTW PinPoint or SRS array for CK plans; all fulfilled the criteria without major discrepancy. Therefore, plans were considered deliverable by both CK and LA-based systems [15].

In the present study, significantly longer treatment times were shown in the CK system. This finding aligns with previous dosimetric comparisons between CK-based robotic radiosurgery and LA-based systems under variable circumstances [30]. Importantly, this finding has pertinent implications for patients whose medical conditions do not allow long irradiation periods, especially those experiencing recurrent VT that leads to life-threatening electrical storms.

Dose limits to heart substructures are largely unknown and have only been recently reported, though not yet correlated to toxicity; however, our institution did not set dosimetry constraints for the coronary arteries. In the present study, as VT substrate could be very close to or overlapping the coronary arteries, the maximal dose of the coronary arteries was as high as 105%-114% of the prescribed dose. The RAVENTA trial has set the major violation for left coronary arteries to be 20 Gy[15]. So far, in our institution, there has been no evidence of short-term toxicities in the coronary arteries. However, patients are followed up after study completion to obtain further long-term safety data. Long-term data will define the full safety profile of cardiac radio-ablation for toxicities on healthy intra-cardiac structures.

Motion management varies between the LA and CK treatment strategies. The internal target volume (ITV) was delineated using four-dimensional CT in both systems to cover respiratory and cardiac movements. Abdominal compression was employed to minimize respiratory motion during the LA-based treatments, which usually required short treatment time with acceptable patient compliance. The advantage of time efficiency in LA-based systems may have biological benefits to treatment outcomes [31] and is also more suitable to life-threatening VT patients experiencing electrical storms. For CK plans with longer treatment times, the CK-based system adopted the synchrony fiducial tracking system (Accuray, Sunnyvale, CA, USA) in which the distal dipole of the right ventricular lead of the implanted cardiac defibrillator is used as a fiducial, allowing real-time tracking of both respiratory and cardiac movements during the long treatment session [12, 13]. Furthermore, the CK-based InCise MLC mode has been used to treat large and irregularly shaped targets with the advantage of shorter delivery time [32], was adopted for patient 12’s treatment plan, and resulted in a relatively acceptable treatment time of 46 min.

The present study had some limitations. First, it only included 13 patients with heterogeneous PTV sizes, shapes, and locations. The small target volumes (PTV ≤ 50 cm^3^) and spherical PTVs were mainly located at the basal segments. However, the large target volumes (PTV > 50 cm^3^) and non-spherical PTVs were mostly located at the apical segments. The small number of patients and heterogeneous PTV volumes, shapes, and locations may limit the comparison of dosimetric advantages between the two systems. Second, the study did not investigate the correlation between dosimetry and clinical outcomes. Further prospective studies with a larger patient size may be needed to validate whether the dosimetric difference affects the clinical efficacy of reducing the VT burden.

## Conclusions

In conclusion, the LA and CK systems are ideal modalities for noninvasive cardiac radio-ablation, with adequate dose coverage and appropriate normal tissue sparing. Moreover, the CK plans provide dosimetric advantages of steeper dose gradients in patients with small target volumes (PTV ≤ 50 cm^3^) and spherical PTVs, although they also require longer treatment times. LA plans may be beneficial in treating large PTVs (> 50 cm^3^) or non-spherical PTVs located at the apical LV lateral wall, with the advantage of time efficiency. Therefore, upfront treatment should be considered based on the clinical intent.

## Data Availability

The data supporting this study’s findings were obtained under license from the National Taiwan University Hospital Healthcare System database and are not publicly available. Data are available from the corresponding author upon request.

## Abbreviations

CI: Conformity index
CK: CyberKnife
CT: Computed tomography
D_2cm_: Maximum dose 2 cm from the planning target volume in any direction
GI: Gradient index
GM: Gradient measure
HI: Homogeneity index
ITV: Internal target volume
LA: Linear accelerator
LV: Left ventricular
OARs: Organs at risk
PIV: Prescription isodose volume
PTV: Planning target volume
SBRT: Stereotactic body radiation therapy
SD: Standard deviation
TG101: American Association of Physicists in Medicine Task Group 101
TPS: Treatment planning system
TV: Target volume
VMAT: Volumetric-modulated arc radiotherapy
VT: Ventricular tachycardia
